# Whole-body magnetic resonance imaging in rheumatology: advancements, key applications and future perspectives

**DOI:** 10.1007/s11547-026-02171-7

**Published:** 2026-02-13

**Authors:** Gabriele Mirabella, Giambattista Privitera, Angelo Montana, Luca Ultimo Esposto, Enzo Fausto Santonocito, Emanuele David, Pietro Valerio Foti, Stefano Palmucci, Placido Romeo, Antonio Basile

**Affiliations:** 1 Department of Medical Surgical Sciences and Advanced Technologies “GF Ingrassia”, University Hospital Policlinico “G. Rodolico-San Marco”, 95123 Catania, Italy; 2 Division of Radiology, San Marco Hospital, AOU Policlinico “G. Rodolico-San Marco”, 95123 Catania, Italy

**Keywords:** Whole-body MRI, Rheumatology, Artificial intelligence, Review, OMERACT

## Abstract

Whole-body magnetic resonance imaging (WB-MRI) is widely used in rheumatology to assess peripheral and axial joints and entheses throughout the body. Despite some limitations, it has potential in determining the overall inflammatory burden, tracking disease progression, and evaluating treatment response. It is used in the evaluation of idiopathic inflammatory myositis (IIM) or in the pediatric population where it is becoming the gold standard for the diagnosis and monitoring of conditions, such as juvenile idiopathic arthritis (JIA) and chronic recurrent multifocal osteomyelitis (CRMO). In addition to advancements in technology and the development of WB-MRI scoring systems, the integration of artificial intelligence (AI) may improve diagnostic accuracy by automating assessments and enabling early detection of subclinical inflammation. The aim of this review is to examine the current scientific evidence for the use of WB-MRI in rheumatology.

## Introduction

Applied mainly in oncologic settings for the detection of bone marrow involvement in malignancies such as multiple myeloma, lymphoma and metastases, whole-body magnetic resonance imaging (WB-MRI) has evolved as a highly valuable diagnostic technique [1, 2]. Today WB-MRI is becoming more common in non-oncological fields, particularly rheumatology, where it provides complete evaluation of multisystem inflammatory disorders [3, 4]. WB-MRI has potential for assessing the complex patterns of systemic inflammation and enthesitis characteristic of rheumatic conditions, including ankylosing spondylitis, psoriatic arthritis, and juvenile idiopathic arthritis [3, 5, 6]. WB-MRI has become popular in pediatric rheumatology for diseases like chronic recurrent multifocal osteomyelitis (CRMO) and juvenile idiopathic arthritis (JIA), where it decreases repeated radiation exposure in long-term follow-up. Moreover, the main advantage of WB-MRI is its ability to identify asymptomatic areas of inflammation that conventional, region-specific imaging techniques may miss [3]. Despite apparent advantages, critical barriers impede widespread clinical translation: absence of prospective randomized controlled trials demonstrating superior diagnostic accuracy or prognostic stratification compared to conventional imaging, absence of economic analyses comparing WB-MRI costs to conventional imaging strategies with documented outcome improvements, radical protocol heterogeneity across institutions preventing meaningful evidence synthesis and lack of standardized scoring systems specific to WB-MRI applications in most rheumatologic conditions [4].

## Methods

This review synthesizes literature, international clinical practice guidelines and recent comprehensive reviews on WB-MRI in rheumatologic diseases spanning 1997 and 2025. Critical gaps in evidence—including absence of prospective randomized trials, health economic analyses, and protocol standardization—were explicitly identified. This approach follows SANRA criteria for quality assessment of narrative reviews [7].

### WB-MRI protocol

Although there is no standard protocol for WB-MRI in rheumatology, usually it consists of T1-weighted and Short Tau Inversion Recovery (STIR) sequences as recommended by the ESSR subcommittee [4]. Giraudo et al. conducted a survey to examine the various protocols employed in Europe: other participants not using such guidelines, applied for example protocols based on the most updated literature, disease-tailored, and the ESTHER trial protocol; 51% of the participants, especially radiologists working in private practice (*p* = 0.025), referred to the guidelines of the Arthritis subcommittee of the ESSR [5, 8].

T1-weighted sequences are essential for the evaluation of structural changes, including fatty infiltration, sclerosis, and erosions in bones. They provide anatomical detail that is critical for the diagnosis of disease progression and chronicity [4, 5, 9]. STIR sequences, on the other hand, are particularly sensitive to fluid and edema, making them ideal for detecting bone marrow edema (BME) and soft tissue inflammation—early indicators of active disease that are critical for timely diagnosis and intervention.

Both 1.5 or 3.0 T scanners can be used equally even though it is shown by Mohan et al. that 1.5 T scanners are preferable in children because 1.5 T has significantly better image quality, fixed structure visibility and fewer artifacts, as compared to WB-MRI at 3 T in children; large studies comparing actual detection of pathology at two field strengths are required [10]. These protocols have been further refined by advancements in WB-MRI technology, which enables scans to be completed in under 45 min without the need to reposition the patient. Modern scanners using numerous phased-array coils—which offer a field of view (FOV) of 15–40 cm along the longitudinal axis—allow complete body scanning in a single session: head, neck, thoracic, abdominal, spine, and lower extremities; the upper limbs are not usually included in the acquisition and need a separate investigation if it is recommended [1].

The whole spine acquisition is advised to be done in sagittal view, STIR/TIRM and/or T1-weighted [4]. For several oncological uses, WB-MRI has become the accepted standard of care imaging method; its non-oncological uses are also growing [1, 11].

Despite not advised as "standard" protocol, there are additional sequences that may be used during WB-MRI, especially Dixon technique and Diffusion-Weighted imaging (DWI). These sequences may improve the sensitivity and specificity of WB-MRI in identifying and characterizing inflammatory processes. The article by Bozgeyik et al. (2008) emphasizes the value of DWI in the early detection of active sacroiliitis, a common symptom of ankylosing spondylitis. This is achieved by measuring apparent diffusion coefficients (ADC) in the sacroiliac joints. Their research revealed that ADC values in affected areas were much greater than in areas with mechanical low back pain, suggesting that DWI is a sensitive and useful technique for spotting early inflammation in sacroiliitis [12]. Using many* b*-values (100, 600, and 1000 s/mm^2^), DWI provides a consistent substitute for contrast-enhanced T1-weighted imaging, which has historically been employed for sacroiliac joint assessment. It also allows separation between acute and chronic inflammatory alterations. Protocol heterogeneity across institutions has prevented standardization of WB-MRI practice, consequently hindering the development of evidence-based clinical guidelines. Major international societies, including the American College of Radiology, have not formally endorsed WB-MRI as a standard imaging modality, with neither the 2017–2022 ACR Appropriateness Criteria for joint pain nor the 2021 update for axial spondyloarthritis providing definitive recommendations for routine clinical use [13].

### Introduction to whole-body MRI in seronegative spondyloarthritis

WB-MRI has emerged as a significant imaging modality for assessing seronegative spondyloarthritis (SpA), a category of chronic inflammatory diseases characterized by a genetic predisposition associated with the HLA-B27 gene and the absence of rheumatoid factor. Classification of spondyloarthritis according to anatomical distribution distinguishes two major phenotypes: axial disease (axSpA), characterized by predominant involvement of the spine and sacroiliac joints, and peripheral disease (pSpA), with primary manifestations in peripheral articulations. In adult populations, ankylosing spondylitis represents the prototypical axial phenotype, whereas psoriatic arthritis, reactive arthritis, enteropathic spondyloarthritis and undifferentiated forms typically present with peripheral joint predominance, though variable axial involvement may develop during disease progression. [14, 15] (Table 1).

**Table 1:** Overview on guidelines and MRI-based scoring system

Disease	Typical population/context	Primary WB‑MRI sites	WB‑MRI hallmarks	Helpful extra sites	Scoring/guidelines	Common pitfalls
Ankylosing spondylitis (axSpA)	Young–middle‑aged; HLA‑B27 often positive	Sacroiliac joints (subchondral BME); spine (vertebral corners)	ASAS‑pattern SIJ BME; Romanus lesions; fat metaplasia → syndesmophytes/ankylosis	Anterior chest wall (MSJ/SCCJ); costovertebral joints	ASAS/OMERACT 2011, EULAR 2015, ACR 2021, ASAS-EULAR 2023; SPARCC SIJ/spine), Berlin/ASspiMRI‑a/‑c, CANDEN, ASAS-SPARTAN 2024	Isolated spinal BME; mechanical stress; infection mimics
Psoriatic arthritis (PsA)	Adults with psoriasis; heterogeneous phenotypes	Hands/feet small joints; SIJs; spine (corners/endplates)	Dactylitis; enthesitis beyond capsule; osteitis; new bone formation	Proximal costal joints; large‑joint entheses	CASPAR; PsAMRIS/HEMRIS; MRI‑WIPE	Overlap with RA/degeneration; distal‑joint resolution on WB‑MRI
Reactive arthritis (ReA)	Post‑infectious; young adults	SIJs; knees/ankles; Achilles/plantar entheses	Peripheral oligoarthritis; insertional BME	Mid‑foot/tarsus	n/a	Imaging overlap with other pSpA—clinical correlation key
Enteropathic SpA (IBD‑related)	Crohn/UC; often young adults	SIJs; hips; variable spine	Bilateral/asymmetric sacroiliitis; enthesitis	Anterior chest wall; pubic symphysis	n/a	Mechanical SIJ change; therapy effects (biologics)
Rheumatoid arthritis (RA)	Adults (F > M);	Wrists, MCP/PIP/MTP; cervical spine (atlanto‑axial)	Synovitis, tenosynovitis, BME, marginal erosions; pannus/retro‑odontoid tissue	Elbows/shoulders (advanced)	2010 ACR/EULAR; RAMRIS (± SAFE/‑5), ERAMRS	Non‑specific synovitis/BME; GBCA improves tenosynovitis; differentiate from PsA
SAPHO	Adults; cutaneous lesions may precede/follow bone disease	Anterior chest wall (SCCJ/MSJ); spine; SIJs	Hyperostosis/osteitis; cortical + medullary change; enthesal sclerosis; juxtaphyseal lesions	Clavicles; sternum; ribs	n/a	Mimics infection/tumor; need small‑FoV SCCJ/chest wall
CRMO/CNO (pediatric CAO spectrum)	Children/adolescents; sterile relapsing–remitting	Metaphyses (tibia), clavicle, pelvis, spine	Multifocal osteitis/BME; subclinical synchronous/metachronous foci; vertebral involvement	Tarsus; sternum	ESSR 2018 Recommendations for multifocal monitoring	Red marrow/open physes mimic edema; confirm with small‑FoV
JIA—ERA/jSpA	Pediatric/teen; male‑predominant; HLA‑B27 frequent	SIJs; thoracolumbar spine; knees/ankles entheses	Early MRI‑only axial inflammation; peripheral enthesitis	Patellar/calcaneal entheses; TMJ (targeted)	ILAR 2001, EULAR 2015, PRINTO 2019, ESSR 2020; pediatric MRI JAMRIS	Age‑related marrow; motion; need targeted TMJ/hands/feet
Polymyalgia rheumatica (PMR)	≥ 50 years; morning stiffness; ↑ESR/CRP	Shoulders/hips/pelvic girdle	SASD bursitis; GH/hip effusions; peritendinitis; extracapsular pelvic edema	Symphysis pubis; acetabular peri‑capsular fat	ACR 2015	Overlap with elderly‑onset RA; avoid over‑calling mild fluid
Systemic sclerosis (SSc)	Adult autoimmune; variable course	Fascia, subcutaneous tissues, muscles; large joints	Fascial/subcutaneous thickening‑edema; perifascial enhancement; myositis‑like edema	Proximal lower limbs; shoulder girdle	EULAR 2023	Chronic fibrosis vs active edema—correlate with CK/clinical
Idiopathic inflammatory myopathies (IIM)	Adults (DM/PM/IBM) or pediatric (JDM)	Proximal limb/girdle muscles; paraspinals	Muscle edema on fluid‑sensitive sequences; fatty replacement; fascial edema	Biopsy planning targets; distribution mapping	EULAR/ACR 2017	Exercise‑related edema; edema vs fat on mapping; motion

*Columns list* typical population/context, primary WB-MRI targets, hallmark findings, helpful extra sites, scoring/guidelines, and common pitfalls* across conditions*

Abbreviations: *WB-MRI*, whole-body magnetic resonance imaging; *axSpA*, axial spondyloarthritis; *AS*, ankylosing spondylitis; *SIJ*, sacroiliac joint; *BME*, bone marrow edema; *MSJ*, manubriosternal joint; *SCCJ*, sternocostoclavicular joint; *MCP/PIP/MTP*, metacarpophalangeal/proximal interphalangeal/metatarsophalangeal joints; *CASPAR*, Classification Criteria for Psoriatic Arthritis; *SPARCC*, Spondyloarthritis Research Consortium of Canada MRI score; *CANDEN*, Canada–Denmark comprehensive spine MRI score; *Berlin/ASspiMRI-a/-c*, Berlin ankylosing spondylitis spine MRI activity/chronicity scores; *RAMRIS*, Rheumatoid Arthritis MRI Score; *ERAMRS*, Extended Rheumatoid Arthritis MRI Score; *PsAMRIS*, Psoriatic Arthritis MRI Score (hands/forefeet); *HEMRIS*, Heel Enthesitis MRI Score; *MRI-WIPE*, Whole-Body MRI Inflammation in Peripheral joints and Entheses (global burden score); *JAMRIS*, Juvenile Arthritis MRI Scoring (WB-MRI module); *ERA*, enthesitis-related arthritis; *JIA*, juvenile idiopathic arthritis; *CRMO/CNO*, chronic recurrent multifocal osteomyelitis/chronic non-bacterial osteomyelitis; *IIM*, idiopathic inflammatory myopathies; *DM/PM/IBM/JDM*, dermatomyositis/polymyositis/inclusion body myositis/juvenile dermatomyositis; *GH*, glenohumeral; *TMJ*, temporomandibular joint; *ACW*, anterior chest wall; *FoV*, field of view

WB-MRI facilitates the identification of inflammation, bone marrow edema, and structural alterations in both axial and peripheral joints, offering a thorough perspective that is especially beneficial for early diagnosis and tracking disease progression. Unlike conventional MRI, it can assess the entire body in a single session, evaluating the multi-district involvement typical of SpA, however, distal small joints may require targeted small-FoV acquisitions to preserve diagnostic detail [13].

### Ankylosing spondylitis

Ankylosing spondylitis (AS) is a chronic inflammatory disease that primarily affects the spine and sacroiliac joints (SIJs) and is the main form of axial spondyloarthritis (axSpA). Early sacroiliitis represents one of the initial observable characteristics of ankylosing spondylitis, and magnetic resonance imaging is essential for identifying these inflammatory changes especially in the non-radiographic form (nr-axSpA), which is not detected at X-ray, but meets clinical criteria for axSpA diagnosis. ASAS–EULAR guidance positions imaging as complementary to clinical/lab assessment: targeted SIJ MRI is preferred for classification/diagnosis. WB-MRI is not recommended routinely and may be considered only for selected whole burden mapping [16].

Bone sclerosis, erosions, and spine ankylosis are also among the primary AS structural findings. One of the imaging markers of AS is Romanus lesions, which manifest as bone marrow edema (BME) at the anterior and posterior vertebral corners. These lesions are frequently a precursor to syndesmophytes, which are bony growths that can eventually result in vertebral fusion and kyphosis as AS advances [17, 18]. Althoff et al. found that 92% of cases of ankylosing spondylitis showed active sacroiliitis as evaluated by whole-body MRI [18]. As previously stated, the addition of Dixon in WB-MRI protocols may facilitate the visualization of acute inflammatory and chronic structural changes, even though it is not a standard protocol [17, 19].

Axial and peripheral enthesitis are hallmark findings in AS. Fourniè et al. reported that inflammatory involvement of the manubriosternal joint occurs in 39–85% of patients with AS. Furthermore, enthesitis of the rotator cuff, characterized by intense acromial bone marrow edema at the deltoid origin, is described as a highly specific feature of AS [20–24]. For standardized reporting/monitoring in axSpA, commonly used MRI scoring systems include SPARCC (SIJ/spine), Berlin, ASspiMRI-a/-c, and CANDEN. [13] A recent International Acquisition Protocol (IAP) for SIJ MRI has been proposed to harmonize planes/sequences and improve reproducibility across: three semi-coronal stacks (T1-weighted, fluid sensitive fat-suppressed, high-resolution structural—3D GRE preferred for erosions) plus one semi-axial fluid sensitive stack, with semi-coronal planning parallel to the dorsal cortex of S2 [25].

### Psoriatic arthritis (PsA)

Psoriatic Arthritis is a chronic inflammatory condition that predominantly impacts axial and peripheral joints, exhibiting an extensive range of musculoskeletal manifestations. This condition is strongly linked to psoriasis and is marked by asymmetric joint involvement; however, it may also present as symmetric polyarthritis, dactylitis-predominant disease, arthritis mutilans, or axial-predominant forms, and in a subset, arthritis can precede cutaneous psoriasis (“psoriatic arthritis sine psoriasis”). The specific diagnosis of PsA is generally confirmed via the 2006 CASPAR criteria [25]. WB-MRI has become an important method in the evaluation of PsA, providing a thorough analysis of synovitis, enthesitis, bone erosions, and new bone formation in both axial and peripheral joints concurrently [1, 26–28].

**Fig.1:**
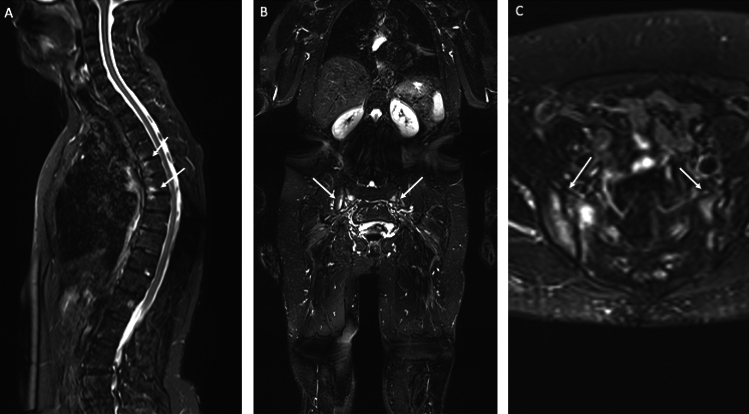
WB-MRI in a 59-year-old woman with PsA. Sagittal Whole-Spine (**a**) show anterior and posterior spondylitis in the thoracic spine. Coronal (**b**) and axial STIR (**c**) planes show bilateral intense sacroiliitis, mostly on the left side

Synovitis is commonly observed in the distal interphalangeal (DIP), metacarpophalangeal (MCP), sacroiliac (SI), and vertebral joints, as indicated by existing literature and our findings [27, 29]. Enthesitis is recognized as a key imaging feature in psoriatic arthritis (PsA) [29–33] (Fig. 1).

All imaging features can be identified using WB-MRI, which provides a comprehensive overview and may assist in determining appropriate treatment. S. Weckbach et al. reported that in their study, about 22 patients (73.3%) had their therapy regimen changed following WB-MRI findings. Specifically, in 18 patients (62.1%), TNF-alpha-inhibitor therapy was initiated based on WB-MRI results, while three patients (10%) moved to methotrexate therapy from prior NSAID medication [29]. WB-MRI may assist in differentiating PsA from other inflammatory arthritis, including rheumatoid arthritis. PsA typically exhibits a distinct pattern of dactylitis and enthesitis, with the enthesitis feature extending significantly beyond the joint capsule and associated with osteitis and synovitis in adjacent tissues. For quantification, MRI scoring systems such as PsAMRIS (hands/forefeet), HEMRIS (heel), and the whole-body MRI-WIPE score (83 peripheral joints and 33 entheses; total 0–738) can be considered [13].

### Rheumatoid arthritis

**Fig. 2:**
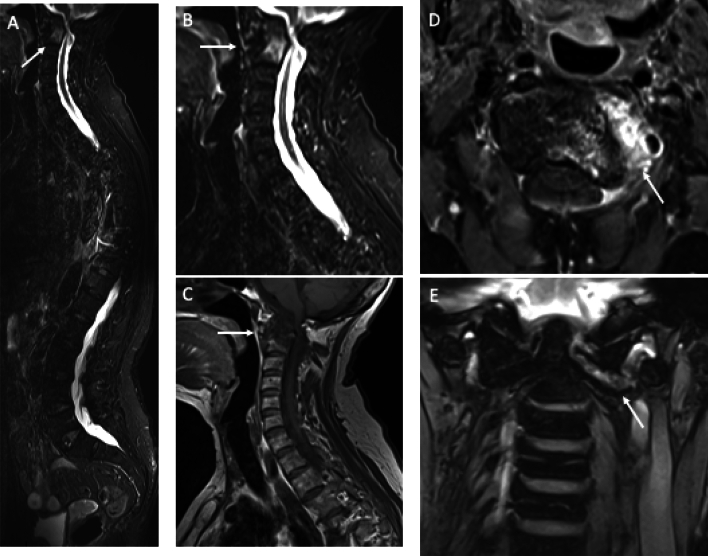
WB-MRI in a 73-year-old woman with atlantoaxial rotatory subluxation due to rheumatoid arthritis (RA). STIR images in sagittal (**a** and **b**) and T1-weighted in sagittal (**c**) planes show that the posterior cortex of the anterior tubercle of C1 and the anterior cortex of the odontoid process are spaced and not parallel. Ax T1 + C (**d**) show intense synovitis. Cor T2 dedicated (**e**) also show spacing

Rheumatoid arthritis (RA) is a systemic autoimmune disorder that predominantly affects synovial joints and tends to have a symmetrical distribution. It frequently affects small joints in the hands and feet. The imaging characteristics of RA encompass synovitis, bone marrow edema (BME), joint effusions, and structural alterations, including erosions and fat infiltration. WB-MRI is beneficial in rheumatoid arthritis due to the common involvement of multiple peripheral joints and the cervical spine. Atlanto-axial level is the most frequent cervical spine location of RA with possible life-threatening complications [34] (Fig. 2). Classification/diagnosis is supported by the 2010 ACR/EULAR criteria (score ≥ 6/10 across joint involvement, serology, acute-phase reactants, and symptom duration), with MRI considered to confirm joint involvement [35] (Table 2).

**Table 2:** Sequence type, coverage, plane, role(s), and comments

Sequence type	Coverage	Plane	Role(s)	Comments
3D T1‑weighted VIBE Dixon	Whole body	Coronal	Anatomic detail/structural overview	Core **T1** required; **Dixon optional**; keep slices consistent (≤ 4 mm WB; thinner for small joints)
STIR (or T2‑FS)	Whole body	Coronal	Inflammatory overview (BME)	Core **fat‑suppressed fluid‑sensitive** sequence (STIR/PD/T2‑FS); STIR preferred for robustness
3D T1‑weighted VIBE Dixon	Whole spine	Sagittal	Anatomic detail	T1 sagittal recommended; 3D optional; harmonize plane & thickness across follow‑ups
STIR (or T2‑FS)	Whole spine	Sagittal	Inflammatory overview (spinal BME)	Pair with T1 sagittal; do not classify axSpA on spine BME alone (SIJ primary)
± DWI / ADC	Whole body	Coronal	Inflammatory overview alternative	Optional, not a substitute for STIR
± STIR (high‑res)	Targeted	Oblique	Detailing of unclear inflammatory foci	Use small‑FoV add‑ons for ACW/SIJ/hands‑feet when WB screen is equivocal
± 3D T1‑weighted VIBE Dixon (thin‑slice)	Targeted	Oblique	Detailing of unclear structural changes	High‑res for erosions/backfill; thinner sections for small joints
± PD‑weighted TSE FS	Targeted	Oblique	Cartilage/tenosynovitis assessment (ACW, peripheral joints)	Peripheral minimum set: T1 + PD/T2‑FS; add post‑contrast T1‑FS for synovitis/tenosynovitis
± 3D T1‑weighted VIBE Dixon, GBCA +	Coronal/Sagittal	Coronal/Sagittal	Enhancement of hypervascular tissues / synovitis	GBCA not routine; reserve for specific differentials or peripheral synovitis/tenosynovitis assessment

Abbreviations: *ADC*, apparent diffusion coefficient; *BME*, bone marrow edema; *DWI*, diffusion-weighted imaging; *FoV*, field of view; *GBCA*, gadolinium-based contrast agent; *PD*, proton density; *SIJ*, sacroiliac joint(s); *STIR*, short-tau inversion recovery; *T2-FS*, T2-weighted fat-suppressed; *TSE*, turbo spin-echo;, Volumetric Interpolated Breath-hold Examination (3D T1-weighted gradient-echo); Dixon, fat–water separation technique (water-only/fat-only reconstructions), WB-MRI, whole-body magnetic resonance imaging

The OMERACT (The Outcome Measures in Rheumatology) MRI Working Group developed and validated the OMERACT RA MRI Scoring system (RAMRIS) from 1998 to 2002. Since then, they updated their recommendations in 2017. They stated that the “core set” of basic MRI sequences in RA recommending T1-weighted images before and after IV gadolinium-contrast injection to better assess the synovitis and T2 fat-saturated or STIR images [36]. They also stated that he implementation of gadolinium (Gd) contrast in MRI for RA substantially improves the detection and evaluation of synovitis and of tenosynovitis by increasing the signal intensity of inflamed tissues; omitting Gd for assessing bone erosions and bone edema has minimal impact on diagnostic accuracy, offering an effective way to decrease costs, invasiveness, and examination duration. However, its accuracy decreases particularly in low-field MRIs, where sensitivity and inter-reader consistency are compromised [36, 37]. Subsequently, in accordance with the low accuracy of low-field MRI, Frenken et al. proposed the RAMRIS-SAFE (Sine-Gadolinium-For-Experts), a GBCA-free procedure for high-field MRI (3T), exhibiting nearly complete concordance with regular RAMRIS scores for synovitis (*ρ* = 0.937) and total joint assessments (*ρ* = 0.976) using STIR sequences [38]. However tenosynovitis detection was less accurate without contrast, according to the Tenosynovitis Scoring System developed by OMERACT group, where they highlighted the importance of post-contrast images to differentiate active tenosynovitis from normal fluid collections [39]. Research suggests that WB-MRI is highly effective in monitoring the response to therapies. Decreases in BME and synovitis scores following treatment are detectable prior to structural changes and are associated with clinical improvement, thereby supporting WB-MRI effectiveness in assessing therapeutic efficacy [40, 41]. However according to the 2023 EULAR management update, imaging (US/MRI) is adjunctive to clinical/lab assessment and routine treatment escalation should not be based solely on imaging, especially in clinical remission; ultrasound is generally first-line for small-joint synovitis, while MRI is reserved for uncertainty or difficult-to-assess areas [42].

### Juvenile idiopathic arthritis (JIA)

**Fig. 3:**
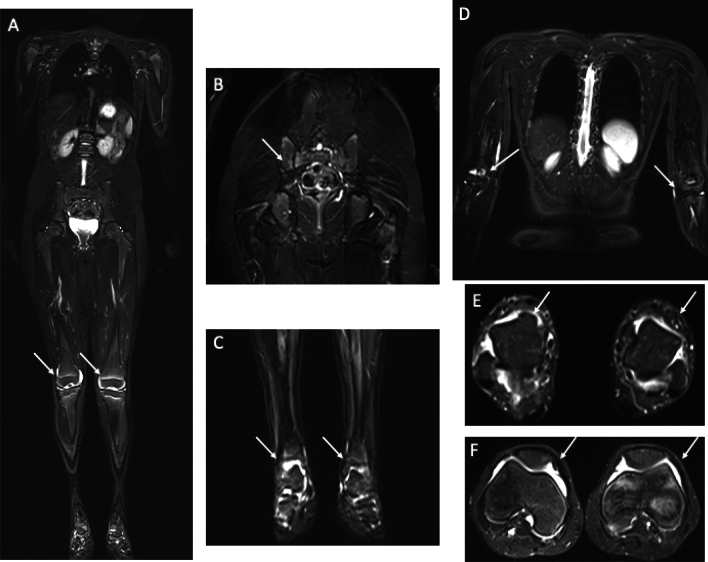
WB-MRI in a 9-year-old girl with oligoarticular juvenile idiopathic arthritis (JIA). Whole-body (**a**) and STIR images in coronal (**b**, **c** and **d**) and axial (**e** and **f**) planes show right sided sacroiliitis (**b**), synovitis in bilateral knees (**a** and **f**), ankles (**c** and **e**) and elbows (**d**) with some spots of BME

Juvenile idiopathic arthritis frequently manifests inflammation in the joints and entheses. The clinical detection of this condition at an early stage can be challenging, particularly in cases of deep-rooted axial joints and pelvic entheses. Such modifications frequently progress without clinical manifestations and can result in irreversible osteochondral joint damage and functional impairments if not addressed [43–45]. The International League of Associations for Rheumatology (ILAR) stratify patients into seven mutually exclusive categories: systemic arthritis (systemic JIA [sJIA]), oligoarthritis, RF-negative polyarthritis, RF-positive polyarthritis, PsA, enthesitis-related arthritis (ERA), and undifferentiated arthritis [46]. The ILAR framework is being revisited, with PRINTO proposing updated categories that include enthesitis/spondylitis-related JIA, largely overlapping with juvenile spondyloarthritis (jSpA) [13] (Fig. 3).

Furthermore, Juvenile Spondyloarthritis (JSpA) refers to a group of inflammatory rheumatological diseases distinct from adult spondyloarthritis where there is an association with the major histocompatibility complex (MHC) class I molecule HLA-B27 in 60–80% of children [47].

Juvenile spondyloarthritis types are mostly represented by the enthesitis-related arthritis (ERA), psoriatic arthritis and undifferentiated arthritis sub-types of JIA [48]. Following PRINTO’s 2019 proposal to refine ILAR categories, “jSpA” designates JIA phenotypes within the spondyloarthritis spectrum—predominantly enthesitis/spondylitis-related JIA (ERA), and in some contexts PsA/undifferentiated forms with enthesitis or early axial disease—whereas “non-jSpA JIA” encompasses the remaining categories (oligoarthritis, RF − /RF + polyarthritis, systemic JIA).

A comprehensive standardized scoring system was developed by a multi-institutional expert panel of radiologists and rheumatologists within the OMERACT in juvenile idiopathic arthritis (JAMRI) working group leading to the creation of a scoring system for WB-MRI called JAMRIS-WB-MRI for inflammation in peripheral, axial joints and entheses [49].

The inter-reader reliability of this scoring system for the evaluation of inflammatory changes proved moderate-to-good results, which may support its use as a standardized evaluation tool in disease characterization and outcome measure studies of joint and enthesis site involvement in JIA.

Choida et al. subsequently published an easy joint assessment system for patients with JIA that was based on post-contrast WB-MRI Dixon images. This system proved that the assessment of multiple sites was feasible and time-efficient in terms of scanning and reporting times [50, 51]. They also demonstrated the value of WB-MRI in identifying JIA by forming two groups: one comprising JIA patients and the other consisting of controls presenting musculoskeletal symptoms but without JIA, to illustrate the efficacy of WB-MRI in detecting disease-specific inflammation. The study demonstrated WB-MRI capacity to distinguish true disease-related inflammation from accidental findings by identifying joint inflammation in 60% of JIA patients, in contrast with just 15% of controls. Moreover, WB-MRI identified subclinical inflammation in over half of the JIA patients [52, 53]. Despite increasing adoption, pediatric WB-MRI has practical (cost, access, scan time) and interpretive limits—distinguishing fluid from synovitis without GBCA, assessing oblique/small joints and immature cartilage, and age-dependent marrow/physis patterns—so reliability improves when whole-body scans are complemented by targeted small-FoV sequences, pediatric expertise, and MRI atlases; formal guidance remains limited for non-jSpA JIA and jSpA. [13]

### Polymyalgia rheumatica (PMR)

Polymyalgia Rheumatica (PMR) is a chronic inflammatory condition predominantly seen in individuals over the age of 50. It is characterized by pain and stiffness in the shoulder and pelvic girdles, along with increased levels of inflammatory markers such as C-reactive protein (CRP) and erythrocyte sedimentation rate (ESR) [54–56]. Mackie et al. found that whole-body MRI can identify a specific subset of PMR patients exhibiting a unique "extracapsular pattern" of inflammation, which correlates with improved responsiveness to glucocorticoid therapy [57]. These Patients, exhibiting inflammation beyond joint capsules and elevated IL-6 levels, demonstrated improved responsiveness to glucocorticoid therapy, highlighting the role of WB-MRI in identifying this subgroup. This discovery may change PMR diagnosis and treatment by providing personalized management strategies. According to Mackie et al., Buttgereit and Matteson highlighted the potential of MRI for improving PMR outcomes by identifying more homogeneous sub-types, guiding glucocorticoid therapy, and informing targeted treatments [58].

However EULAR/ACR 2015 emphasize that PMR is a clinical diagnosis, with imaging used as an adjunct mainly in atypical/uncertain cases or to exclude mimics, where routine MRI/WB-MRI is not recommended by EULAR/ACR and ultrasound is the preferred imaging tool under study for assessment/monitoring [59].

### Chronic recurrent multifocal osteomyelitis (CRMO)

Chronic Recurrent Multifocal Osteomyelitis, also known as Chronic Non-Bacterial Osteomyelitis (CNO), is a rare autoinflammatory bone disorder primarily observed in children and adolescents. It typically involves the metaphyses of long bones, clavicles, and the spine. The typical MRI finding is a hyperintense geographic metaphyseal lesion located near the growth plates of the long bones in the lower extremities, as well as potential lesions in the spine, pelvis, clavicle, and/or sternum [60–62]. WB-MRI serves as the primary diagnostic method for identifying both symptomatic and asymptomatic lesions, monitoring disease progression, and CRMO from infections or malignancies.

Andronikou et al. identified three patterns: (1) a tibioappendicular multifocal pattern characterized by multifocal appendicular lesions with predominant tibial involvement; (2) a claviculospinal pauci-focal pattern, primarily involving clavicular lesions with few others, mainly in the spine; and (3) a tibioclavicular crossover pattern, featuring simultaneous involvement of both clavicular and tibial regions [63]. Astolfi et al. supported, in according to other studies, that the inclusion of DWI in the protocol due to its potential for high sensitivity in detecting bone marrow edema foci and its possible utility in distinguishing malignancy from chronic recurrent multifocal osteomyelitis (CRMO) in the spine but it is not universally accepted [60, 64, 65].

**Fig. 4:**
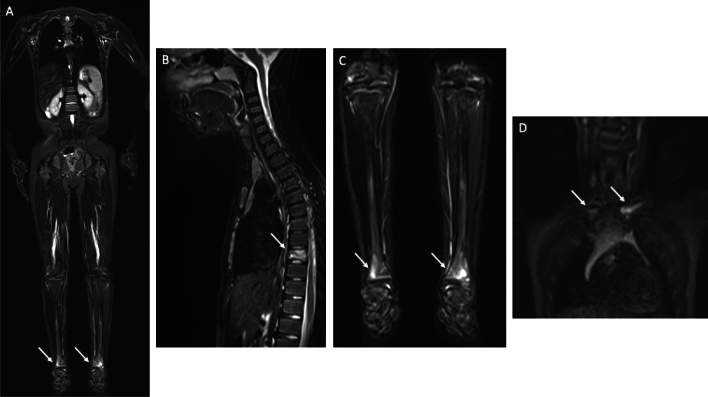
WB-MRI in a 9-year-old boy with CRMO. WB STIR (**a**) and Cor STIR (**c**) plane images show bilateral BME of the meta-epiphyseal distal tibia. Sagittal STIR (**b**) plane image show a fracture of the D9 vertebral body. Cor STIR (**d**) plane show BME of the sternal end of bilateral clavicle, mostly on the left side

Merlini et al. demonstrated in their study that DWI did not enhance lesion conspicuity in comparison with STIR [66]. WB-MRI allows early diagnosis, reduces the need for invasive biopsies, and is essential for evaluating treatment responses and complications such as vertebral deformities, where early vertebral involvement should be actively screened to prevent height loss/vertebra plana. Age-related red marrow and growth-plate signal can mimic edema—small-FoV spine/SIJ sequences improve specificity, while a pragmatic WB-MRI setup centers on coronal STIR with targeted sagittal spine/knees/ankles and axial pelvis as needed [67, 68] (Fig. 4).

### SAPHO syndrome (synovitis, acne, pustulosis, hyperostosis, and osteitis)

SAPHO syndrome is an uncommon autoinflammatory disorder that is distinguished by a combination of skin and musculoskeletal manifestations, including synovitis, acne, pustulosis, hyperostosis, and osteitis. SAPHO and its pediatric counterpart (CRMO/CNO) can be framed within the chronic aseptic osteitis (CAO) spectrum, with “incomplete” variants such as sternocostoclavicular hyperostosis and pustulotic arthro-osteitis. [13] SAPHO in children and adolescents mainly affects long bone metaphyses, with clavicles being the next most involved sites. In adults, it predominantly affects the anterior chest wall, particularly the sterno-costo-clavicular junction, followed by the axial skeleton, including the spine and sacroiliac joints [69, 70]. The standard protocol for WB-MRI, whether with or without T1 post-gadolinium sequences, has been proved to be beneficial in identifying subclinical occult sites, which are identified as areas of bone marrow edema [69, 71]. Small-FoV targeted acquisitions (e.g., sternocostoclavicular region and other symptomatic sites) help preserve spatial detail and interpretability.

Fritz et al. showed that 67% of lesions identified on whole-body MRI were totally asymptomatic [72]. Chianca et al. demonstrated, according to Hidetomo et al., that whole-body MRI exhibits greater sensitivity than bone scintigraphy in identifying juxtaphyseal lesions [1, 73].

### Myositis

**Fig. 5:**
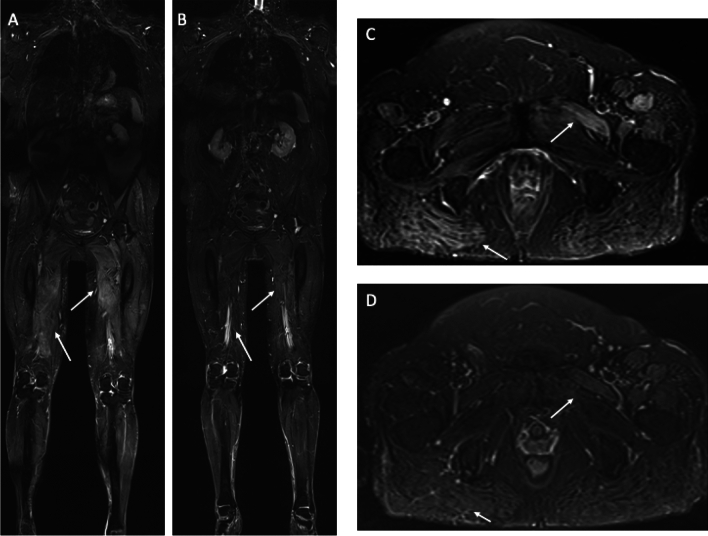
WB-MRI of a 68-year-old woman with necrotizing myopathy. Image in **a** and **c** show diffuse muscle edema on the coronal and axial STIR planes. WB-MRI performed as follow-up 2 months later after starting treatment and stopping statin demonstrated a partial resolution of the muscle edema (coronal and axial STIR in **b** and **d**). Pathological anatomy requested a search for Anti-HMG-CoA reductase antibodies on suspicion of statin-induced necrotizing autoimmune myopathy (SINAM)

Myositis defines a group of idiopathic inflammatory myopathies (IIM) marked by muscular inflammation, typically manifesting as symmetrical proximal muscle weakness, increased muscle enzymes, and distinctive histopathological characteristics [74–76]. The most common sub-types include dermatomyositis (DM), polymyositis (PM), immune-mediated necrotizing myopathy (IMNM), overlap myositis (OM) with the subgroup of anti-synthetase syndrome (ASS), sporadic inclusion body myositis (sIBM), inflammatory myopathy with mitochondrial pathology (IM-Mito) and statin-induced necrotizing autoimmune myopathy (SINAM), all of which are infrequent disorders that frequently pose diagnostic difficulties and present significant management challenges [77–79] (Fig. 5). WB-MRI has become an invaluable tool in the diagnosis and monitoring of these conditions, as it offers comprehensive insights into the distribution and severity of muscle involvement, frequently surpassing other diagnostic modalities [80, 81]. As an example, Huang et al. concluded that WB-MRI had a higher positive rate than the serum creatine kinase test and EMG in the role of diagnosis [77, 82] Essouma et al. claim in their scoping review the intention to create pediatric and adult whole-body/dedicated MRI scoring systems for the diagnosis, evaluation, and monitoring of idiopathic inflammatory myopathy-related skeletal muscle damage in both research and clinical settings [83]. MRI may also assist in the selection of the optimal biopsy site by identifying the highest inflammatory activity with the STIR sequence, which decreases the false negative rate. This is necessary for the definitive diagnosis of an inflammatory myopathy, as microscopic examination of the muscle biopsy is essential. Additionally, MRI could confirm the diagnosis and rule out any other causes of myopathy [84].

Moreover, WB-MRI is an essential instrument for evaluating the overall inflammatory burden in Juvenile Dermatomyositis (JDM), a rare autoimmune disorder characterized by symmetrical proximal muscle weakness, chronic inflammation of the skin and skeletal muscles, and systemic involvement that may result in permanent disability. It identifies inflammation in both symptomatic and asymptomatic regions, covering subcutaneous tissue and myofascia [85]. Malattia et al. additionally highlight WB-MRI observations regarding disease activity and patterns of muscle inflammation (e.g., patchy vs diffuse) [86]. However in current EULAR/ACR classification, MRI is supportive but not mandatory; diagnosis remains clinico-serologic with biopsy when indicated [87].

### Systemic sclerosis

Systemic sclerosis (SSc) is a chronic autoimmune disorder distinguished by progressive fibrosis caused by the excessive accumulation of extracellular matrix components in many tissues and organs. Vascular damage, inflammation, and the presence of specific autoantibodies are also suggestive of SSc [88, 89].

Schanz et al. focused on the characterization of musculoskeletal manifestations through whole-body MRI, which facilitated the recording of their distribution. The proximal regions of both extremities are believed to be the muscular sites that are preferentially affected by SSC.

Fasciitis, synovitis, tenosynovitis, and enthesitis are frequently observed, with a symmetrical distribution [90, 91]. EULAR 2023 recommendations frame imaging as adjunctive and do not recommend routine WB-MRI in SSc; MRI should be targeted to specific clinical questions (e.g., suspected myopathy or tendon involvement) rather than used systematically [92].

### Avascular multifocal osteonecrosis (AMO)

Avascular multifocal osteonecrosis is an uncommon but debilitating syndrome that is distinguished by the death of bone tissue at multiple sites because of disrupted blood flow. AMO frequently impacts individuals with autoimmune or hematologic disorders undergoing high-dose corticosteroid therapy, potentially resulting in substantial joint and bone abnormalities if not appropriately detected and controlled. Yokota. et al. examined WB-MRI and whole-body bone scintigraphy (WB-BS) for the identification of multifocal osteonecrosis (ON); they found that ON is asymptomatic prior to collapse and is distinguished by a band-like pattern in T1 images. It becomes symptomatic and painful following the collapse due to bone marrow edema and joint effusion. WB-BS shows a lower degree of agreement with WB-MRI findings and has difficulty detecting asymptomatic ON, particularly in joints outside the hip [93, 94]. In accordance with other studies, they demonstrated that WB-MRI has a great sensitivity and accuracy, allowing the identification of early stage osteonecrosis and multifocal lesions in many joints, including the hip, knee, ankle, and shoulder [95, 96].

### Artificial intelligence in whole-body MRI for rheumatology

Artificial intelligence (AI) has the potential to significantly influence the future of WB-MRI applications in rheumatology. Studies show that AI plays a significant role in the early detection of synovitis, BME, and bone erosions in these two rheumatic diseases, as evidenced by multiple studies [97–102]. Chandrika et al. performed a pilot study on CNO treated with pamidronate, using AI to evaluate pre- and post-treatment whole-body MRI scans for therapeutic response. A machine learning algorithm was developed to classify lesions as improved, worsened, or stable, with outcomes compared to those of expert radiologists. The AI model achieved a high level of sensitivity with 100% accuracy; however, it exhibited low specificity, misclassifying stable lesions, and resulting in an overall accuracy of 33.3%. This limitation was attributed to the challenge of distinguishing genuine pathological changes from normal variations or anomalies, as well as the small dataset size and the focus on restricted regions such as the knee. The integration of AI with WB-MRI has the potential to improve clinical practice by employing larger datasets, refining algorithms, and increasing coverage, which will allow more accurate and reliable assessments of treatment responses for CNO and other multifocal diseases [103].

However, other important critical limitations must be acknowledged: deep learning models are particularly prone to overfitting on small datasets, with 81% of published external validation studies showing diminished algorithm performance when applied to different institutions, and demographic biases in training data can compromise accuracy across diverse patient populations [104, 105].

## Conclusions

WB-MRI is a powerful one-stop technique to phenotype inflammatory arthritis and quantify global disease activity, with growing support from initiatives that aim to harmonize acquisition, scoring, and reporting.

Its clearest clinical role today is in pediatrics—particularly CNO/CRMO, where WB-MRI helps establish diagnosis, map burden (including vertebral disease), and monitor response while avoiding radiation. In adults, WB-MRI can add value in selected scenarios (complex or multisite phenotypes; discordant clinical–imaging findings; suspected anterior chest wall or extra-axial disease; trial baselines), but routine use is not yet evidence-based given limited prospective comparative data, protocol heterogeneity, and uncertain health-economic impact. Until these gaps are addressed, a pragmatic approach is to reserve WB-MRI for well-defined indications (e.g., pediatric disease, radiation-sparing strategies, multisystem burden mapping, research/treat-to-target cohorts), interpret findings in clinical context, and standardize reporting with validated MRI scores to maximize reproducibility and clinical utility.
